# Interventionen gegen BK-Virusinfektionen bei Empfängern von Nierentransplantaten

**DOI:** 10.1007/s00120-024-02491-4

**Published:** 2024-12-23

**Authors:** Laila Schneidewind

**Affiliations:** Universitätsklinik für Urologie, Inselspital Bern, Universität Bern, Freiburgstr. 37, 3010 Bern, Schweiz

## Originalpublikation

Wajih Z, Karpe KM, Walters GD (2024) Interventions for BK virus infection in kidney transplant recipients. Cochrane Database of Systematic Reviews. 10.1002/14651858.CD013344.pub2.

## Übersetzung

### Hintergrund.

Die BK-virusassoziierte Nephropathie (BKVAN), die durch eine Infektion mit dem BK-Virus oder dessen Reaktivierung verursacht wird, ist nach wie vor eine Herausforderung bei der Nierentransplantation. Das Screening wird für alle Personen, die eine Niere transplantiert bekommen, empfohlen. Bei Patient*innen mit klinisch signifikanter Infektion stellt die Verringerung der Immunsuppression den zentralen Ansatz der Behandlung dar. Es gibt keine spezifische antivirale oder immunmodulatorische Therapie, die ausreichend wirksam für den routinemäßigen Einsatz ist.

### Ziele.

Ziel dieser Übersichtsarbeit war es, den Nutzen und Schaden von Maßnahmen zur Behandlung der BK-Virusinfektion bei Nierentransplantatempfängern zu untersuchen.

### Suchmethodik.

Wir durchsuchten das Cochrane Kidney and Transplant Register of Studies bis zum 5. September 2024 durch Kontakt mit Informationsspezialisten unter Verwendung von Suchbegriffen, die für diesen Review relevant sind. Die in das Register aufgenommenen Studien werden durch Recherchen in CENTRAL, MEDLINE und EMBASE, in Konferenzberichten, dem Suchportal der International Clinical Trials Registry Platform (ICTRP) und in ClinicalTrials.gov identifiziert.

### Auswahlkriterien.

Alle randomisierten kontrollierten Studien (RCT) und Kohortenstudien, die eine Intervention zur Behandlung oder Prävention von BKVAN bei Empfänger*innen von Nierentransplantaten untersuchten, wurden berücksichtigt.

### Datensammlung und Datenanalyse.

Zwei Autor*innen bewerteten unabhängig voneinander die Qualität der Studien und extrahierten die Daten. Zusammenfassende Schätzungen des Effekts wurden mithilfe eines Random-effekts-Modells ermittelt, und die Ergebnisse wurden als Risikoverhältnisse (RR) und deren 95 %-Konfidenzintervalle (KI) für dichotome Ergebnisse und als mittlere Differenz (MD) und 95 %-KI für kontinuierliche Ergebnisse dargestellt. Das Vertrauen in die Evidenz wurde anhand des GRADE-Ansatzes („grading of recommendations assessment, development and evaluation“) bewertet.

### Hauptergebnisse.

Zwölf RCT mit 2669 randomisierten Teilnehmenden wurden eingeschlossen. Sechs Studien wurden in einzelnen Zentren durchgeführt, 6 waren multizentrische Studien; 2 davon waren internationale Studien. Die Altersspanne der Teilnehmenden reichte von 44 bis 57 Jahren. Die Dauer der Nachbeobachtung reichte von 3 Monaten bis zu 5 Jahren. Alle Studien schlossen Menschen mit einer Nierentransplantation ein, 3 Studien schlossen Menschen mit Anzeichen einer BK-Virämie ein. Die Studien waren in Bezug auf die Art der Maßnahmen und die bewerteten Endpunkte heterogen. Das allgemeine Risiko einer Verzerrung war niedrig oder unklar.

Ein intensives Screening zur frühzeitigen Erkennung von BK-Virämie oder BK-Virurie verhindert den Transplantatverlust (1 Studie, 908 Teilnehmende: RR 0,00, 95 %-KI 0,00 bis 0,05) und verringert das Vorhandensein von Decoy-Zellen und Virämie nach 12 Monaten (1 Studie, 908 Teilnehmende: RR 0,06, 95 %-KI 0,03 bis 0,11) im Vergleich zur Routineversorgung (Evidenz von hoher Vertrauenswürdigkeit). Andere Ergebnisse wurden nicht berichtet.

Im Vergleich zu Placebo verringern Fluorchinolone möglicherweise das Risiko eines Transplantatverlustes geringfügig (3 Studien, 393 Teilnehmende: RR 0,37, KI 0,09 bis 1,57; I2 = 0 %; Evidenz von niedriger Vertrauenswürdigkeit), beeinflussen wahrscheinlich nicht oder nur geringfügig spenderspezifische Antikörper (DSA), beeinflussen möglicherweise nicht oder nur geringfügig BK-Virämie und Tod. Im Vergleich zu Placebo ist die Wirkung von Fluorchinolonen auf BKVAN und Malignität ungewiss, möglicherweise erhöhen Fluorchinolone aber das Risiko einer Sehnenscheidenentzündung (2 Studien, 193 Teilnehmende: RR 5,66, KI 1,02 bis 31,32; I2 = 0 %; Evidenz von niedriger Vertrauenswürdigkeit).

Im Vergleich zu Tacrolimus (TAC) beeinflusst Cyclosporin (CSA) wahrscheinlich nur geringfügig oder gar nicht Transplantatverlust und Tod, beeinflusst möglicherweise nur geringfügig oder gar nicht BKVAN und Malignität, verringert aber wahrscheinlich die BK-Virämie (2 Studien, 263 Teilnehmende: RR 0,61, 95 %-KI 0,26 bis 1,41; I2 = 38 %) und reduziert wahrscheinlich das Risiko eines neu auftretenden Diabetes nach der Transplantation (1 Studie, 200 Teilnehmende: RR 0,41, 95 %-KI 0,12 bis 1,35; für beide Endpunkte Evidenz von moderater Vertrauenswürdigkeit).

Im Vergleich zu Azathioprin hat Mycophenolat-Mofetil (MMF) wahrscheinlich keinen oder nur einen geringen Einfluss auf den Transplantatverlust und die BK-Virämie, verringert aber wahrscheinlich das Risiko für Tod (1 Studie, 133 Teilnehmende: RR 0,43, 95 %-KI 0,16 bis 1,16) und Malignität (1 Studie, 199 Teilnehmende: RR 0,43, 95 %-KI 0,16 bis 1,16; für beide Endpunkte Evidenz von moderater Vertrauenswürdigkeit).

Im Vergleich zu Mycophenolat-Natrium (MPS) ist die Wirkung von CSA auf Transplantatverlust und Tod ungewiss, beeinflusst CSA möglicherweise die BK-Virämie nicht oder nur geringfügig, kann aber möglicherweise BKVAN reduzieren (1 Studie, 224 Teilnehmende: RR 0,06, 95 %-KI 0,00 bis 1,20; Evidenz von niedriger Vertrauenswürdigkeit).

Im Vergleich zu einer Dosisreduzierung der Immunsuppression beeinflusst die Umstellung von MMF oder TAC auf Everolimus oder Sirolimus möglicherweise Transplantatverlust, BK-Virämie oder BKVA nicht oder nur geringfügig (Evidenz von niedriger Vertrauenswürdigkeit). Die Umstellung von TAC auf Sirolimus führt wahrscheinlich bei mehr Menschen zu einer reduzierten BK-Viruslast (< 600 Kopien/ml) als eine Reduzierung der Immunsuppression (1 Studie, 30 Teilnehmende: RR 1,31, 95 %-KI 0,90 bis 1,89; Evidenz von moderater Vertrauenswürdigkeit).

Im Vergleich zu MPS ist die Wirkung von Everolimus auf Transplantatverlust und die BK-Virämie ungewiss, möglicherweise verringert Everolimus aber BKVAN (1 Studie, 135 Teilnehmende: 0,06, 95 %-KI 0,00 bis 1,11) und erhöht möglicherweise das Sterberisiko (1 Studie, 135 Teilnehmende: RR 3,71, 95 %-KI 0,20 bis 67,35; für beide Endpunkte Evidenz von niedriger Vertrauenswürdigkeit).

Im Vergleich zu CSA beeinflusst Everolimus möglicherweise nur geringfügig oder gar nicht BK-Virämie, hat ungewisse Wirkungen auf den Transplantatverlust und BKVAN, erhöht aber möglicherweise das Sterberisiko (1 Studie, 185 Teilnehmende: RR 3,71, 95 %-KI 0,42 bis 32,55; Evidenz von niedriger Vertrauenswürdigkeit).

Das Leflunomid-Derivat FK778 hat im Vergleich zur Reduktion der Immunsuppression möglicherweise nur einen geringen oder gar keinen Einfluss auf den Transplantatverlust, führt wahrscheinlich zu einer stärkeren Senkung der BK-Viruslast im Plasma (1 Studie, 44 Teilnehmende: −0,60 Kopien/µl, 95 %-KI −1,22 bis 0,02; Evidenz von moderater Vertrauenswürdigkeit), hat aber ungewisse Wirkungen auf BKVAN und Malignität. Hoher Blutdruck kann durch KF778 verstärkt werden (1 Studie, 46 Teilnehmende: RR 8,23, 95 %-KI 0,50 bis 135,40; Evidenz von niedriger Vertrauenswürdigkeit). In beiden Gruppen gab es keine Todesfälle.

### Schlussfolgerung der Autoren.

Eine intensive Überwachung auf BK-Virurie und BK-Virämie in der Frühphase nach der Transplantation ist für die Verbesserung der Ergebnisse bei BK-Virusinfektionen von großer Bedeutung. Sie ermöglicht eine frühzeitige Infektionserkennung und rechtzeitige Anpassung der Immunsuppression. Es gibt keine ausreichende Evidenz für andere Maßnahmen zur Behandlung der BK-Virusinfektion bei Nierentransplantatempfänger*innen.

## Kommentar

Bei Empfängern von Nierentransplantaten ist die Reaktivierung des BK-Virus häufig. Die Reaktivierung ist allerdings meist subklinisch, obwohl sie sich als akute Nierenschädigung manifestieren kann und ein wesentlicher Risikofaktor für den vorzeitigen Verlust des Transplantats ist. Die BK-virusassoziierte Nephropathie (BKVAN) ist eine bedeutende Ursache für Nierentransplantatversagen und betrifft etwa 1–10 % der Patienten. Daher wird ein Screening auf Reaktivierung des Virus für alle Nierentransplantierten empfohlen. Es gibt keine spezifische antivirale oder immunmodulatorische Therapie, die für den Routineeinsatz ausreichend wirksam ist. Mit anderen Worten, bis zum aktuellen Zeitpunkt steht keine kausale Behandlung sowohl für die Virus-Reaktivierung als auch für die Infektion zur Verfügung. Aus diesem Grund ist bei Patienten mit klinisch signifikanter Reaktivierung die Reduktion der Immunsuppression der Eckpfeiler der Therapie [1, 2]. Zu den Risikofaktoren für eine BK-Virusinfektion gehören Leukozytenantigen (HLA)-Mismatch, Postmortemspende, weibliche Spender und männliche Empfänger, älteres Alter der Empfänger, akute Abstoßungsepisoden und die Art der immunsuppressiven Medikation [1, 3]. Daraus muss konsequenterweise geschlussfolgert werden, dass es sich bei dem vorliegenden Cochrane Review um eine essenzielle Arbeit zur Verbesserung der Ergebnisse der Nierentransplantation handelt.

Unglücklicherweise ergibt sich aber kaum Evidenz für eine bestimmte Therapie. Lediglich Fluorchinolone können das Risiko eines Transplantatverlusts leichtgradig verringern, doch auch hier gibt es nur einen geringen Evidenzlevel. Hierzu muss angemerkt werden, dass bei Einsatz von Fluorchinolonen aufgrund der Gefahr eines Kollateralschadens sowie der bakteriellen Resistenzentwicklung eine stringente Nutzen-Risiko-Einschätzung vorgenommen werden muss [4]. Damit bleibt die intensive Überwachung auf BK-Virurie und Virämie nach der Nierentransplantation aktuell der wichtigste Teil im Management der BKVAN, denn diese ermöglicht ein frühes Reagieren auf die Virusreplikation und nachfolgend ggf. die Reduktion der Immunsuppression. Zu dem gleichen Schluss kommt die zweite internationale Konsensus-Leitlinie zum Management des BK Polyomavirus in der Nierentransplantation. Es wird dabei keine Empfehlung für den Gebrauch von Leflunomid, Cidofovir, Fluorchinolonen oder Immunglobulinen ausgesprochen. Weiterhin weisen die Autoren dieser Leitlinie daraufhin, dass ein erheblicher Forschungsbedarf in diesem Bereich besteht, insbesondere robuste randomisierte klinische Studien (RCT) fehlen. Neue Verfahren bzw. Therapien gegen BKVAN, wie z. B. neue Virusstatika, neutralisierende Antikörper, spezifische T‑Zellen und Immunglobuline, sollten in diesen RCT getestet werden. Außerdem muss diskutiert werden, dass die vorliegende Leitlinie ebenfalls sehr aktuell ist und weitere wesentliche Aspekte der Thematik beleuchtet, die in dem vorliegenden Cochrane Review nicht adressiert werden. Es werden Empfehlungen zur pädiatrischen Nierentransplantation abgegeben, während in der vorliegenden Übersichtsarbeit keine pädiatrischen Patienten eingeschlossen worden sind. Allerdings ist die differenzierte Betrachtung hier essentiell, da bei Kindern und Jugendlichen andere pharmakokinetische und -dynamische Eigenschaften zu berücksichtigen sind. Zusätzlich sind in dieser Leitlinie noch die Diagnostik sowie Kosten-Nutzen und Kosten-Risiko-Betrachtungen inkludiert [3].

Trotzdem ist das vorliegende Cochrane Review methodisch robust und schlussendlich kann Evidenz nur aus soliden eingeschlossen Studien resultieren. So ist z. B. der Zeitpunkt der Untersuchungen auf BKVAN in den inkludierten Studien zu diskutieren. Laut dem europäischen Textbuch für Nierentransplantation ist das Auftreten für BKVAN nach dem ersten Monat bis zum sechsten Monat nach der Transplantation am häufigsten [5]. Dies verdeutlicht den Fakt, wie wichtig eine detaillierte Studienplanung für einen robusten RCT ist. Weiterhin ist das vorgestellte Cochrane Review sehr aktuell, da die letzte Suche am 5. September 2024 durchgeführt worden ist. Allerdings konnten in einer Update-Suche am 03. November 2024 zwei weitere Studien identifiziert werden, die die Einschlusskriterien erfüllen. Chandraker et al. führten eine doppelblinde, placebokontrollierte, randomisierte Phase-II-Studie zu Posoleucel, einem allogenen multivirusspezifischem T‑Zell-Konstrukt, in adulten nierentransplantierten Patienten mit BK-Virämie durch. Die Autoren kommen zu dem Schluss, dass Posoleucel i. Allg. sicher und gut verträglich ist und im Vergleich zu Placebo zu einer stärkeren Verringerung der BK-Virämie führt. Zu den Einschränkungen dieser Studie gehören die relativ kurze Dauer der Nachbeobachtung und die Tatsache, dass sie unterpowert war, um signifikante Unterschiede bei den klinischen Ergebnissen festzustellen [6]. Imlay et al. führten ebenfalls eine randomisierte, placebokontrollierte Dosiseskalations-Phase-I/II-Studie bei Erwachsenen mit BKVAN mit niedrig dosiertem Cidofovir durch. Die Studie wurde wegen der langsamen Rekrutierung vorzeitig abgebrochen, nachdem 22 Nierentransplantierte die Studie abgeschlossen hatten [7]. Somit ergibt sich mit den 2 neu publizierten Studien keine Änderung der Evidenzlage.

Im vorliegenden Cochrane Review werden zwar lediglich Erwachsene betrachtet, doch der Vollständigkeit halber muss auch eine aktuelle Arbeit zur pädiatrischen Nierentransplantation genannt werden. Aksoy et al. untersuchten retrospektiv 36 pädiatrische, nierentransplantierte Patienten mit der Frage, welches Virostatikum (Leflunomid vs. Cidovofir) in der Therapie der BKVAN besser sei. Die Ergebnisse waren ähnlich zwischen der Cidofovir- und Leflunomid-Gruppe hinsichtlich des Kreatininspiegel bei der letzten Nachuntersuchung (0,91 ± 0,29 vs. 0,94 ± 0,37 mg/dl; *p* = 0,843) und des Transplantatversagen (8,3 % bzw. 14,2 %; *p* = 0,632). Ein Transplantatversagen wurde bei 8,3 % der Patienten mit BKVAN beobachtet. Daher kamen die Autoren zu der Schlussfolgerung, dass Leflunomid und Cidovofir ähnliche Ergebnisse bei der Behandlung der BKVAN bei Kindern erzielen [8]. Doch auch hier ist der Evidenzgrad aufgrund der kleinen Fallzahl und des retrospektiven Studiendesigns als gering zu bewerten. Getrennte solide Studien und Metaanalysen sind für diese spezielle Population absolut essenziell und wünschenswert.

Ein klinisch orientierter Cochrane Review kann es zwar nicht leisten, experimentelle Studien zusammen zu stellen und zu bewerten, aber eine systematische Übersichtsarbeit zu experimentellen Arbeiten über neue therapeutische Ansätze bzw. neue Substanzen wäre ebenfalls erstrebenswert, da so erste klinische Phase-I-Studien besser geplant werden könnten. Außerdem sei erwähnt, dass es eine weitere wichtige Patientengruppen gibt, die unter BK-assoziierten Erkrankungen, wie z. B. der hämorrhagischen Zystitis, leidet und für die ebenfalls keine kausale Therapie zur Verfügung steht. Dies sind die allogen stammzelltransplantierten Patienten [9].

Abschließend muss betont werden, dass das wichtigste Ergebnis des Cochrane Reviews die Implikationen für die weitere Forschung ist. Die Autoren kommen zu folgender finaler Aussage: Daten aus RCT bzgl. Interventionen zu BK-Virurie, BK-Virämie und BKVAN in Hinblick auf die Transplantatgesundheit werden dringend benötigt. Wie bereits oben erläutert, kommt die aktuelle Konsens-Leitlinie zum Management des BK-Virus bei Nierentransplantierten zu dem gleichen Ergebnis [3]. Zusammenfassend werden also dringend gut geplante, robuste RCT sowohl bei Erwachsenen als auch bei Kindern und Jugendlichen benötigt, um die Ergebnisse der Nierentransplantation weiter verbessern zu können.

## Fazit für die Praxis

- Es gibt weiterhin keine wirksame kausale Therapie der BKVAN bei adulten Nierentransplantierten.
- Eckpfeiler des Managements der BKVAN ist weiterhin das leitliniengerechte Screening sowie dann ggf. die frühzeitige Reduktion der Immunsuppression.
- Es werden dringend robuste, detailliert geplante RCT mit adäquater Power benötigt – nur so können auch neue Substanzen solide bewertet werden.
- Kinder und allogen Stammzelltransplantierte müssen in RCT ebenfalls dringend untersucht werden, eine getrennte Betrachtung ist hierbei sinnvoll.
